# Something’s different: elaboration’s transferrable role for false alarm reduction

**DOI:** 10.1186/s41235-025-00623-8

**Published:** 2025-03-18

**Authors:** Lauren A. Mason, Abigail Miller, Gregory Hughes, Holly A. Taylor

**Affiliations:** 1 Tufts University, 490 Boston Ave., Medford, MA 02155 USA; 2 U.S. Army DEVCOM Soldier Center, 15 General Greene Ave., Natick, MA 01760 USA; 3 Center for Applied Brain and Cognitive Sciences, Tufts University, 177 College Ave., Medford, MA 02155 USA

**Keywords:** Error detection, False alarm, Elaboration

## Abstract

False alarming, or detecting an error when there is not one, is a pervasive problem across numerous industries. The present study investigated the role of elaboration, or additional information about non-error differences in complex visual displays, for mitigating false error responding. In Experiment 1, learners studied errors and non-error differences about a virtual LEGO® model. Half of the participants received information about the error (location, omission, orientation) and difference (color, addition) categorization and identification (i.e., what constituted the error or difference). The other half of participants received the same information plus further elaboration about (1) the potential consequences of errors and (2) why differences would not pose potential problems. Receiving additional elaboration about errors and differences aided learners’ ability to accurately reject non-error differences at test. Experiment 2 replicated these results with a new stimulus model and extended findings by testing whether receiving elaboration on the first model transferred to support learning in a second, similar model that did not provide elaborations. Our results replicated and extended findings from Experiment 1, such that learners who received elaboration while learning the first model also performed better at correctly rejecting non-error differences at test on the second model. Taken together, our findings provide insight on the transferrable role of feature elaboration to reduce false alarm rates during complex visual display assessments.

## Introduction

Imagine you are a soldier checking a payload containing food and medical aid for assembly errors before it is dropped via parachute from an airplane. High-stakes situations that require professionals to efficiently and effectively detect errors bridge many industries. Outside of military and first responder contexts, medical practitioners must review images to detect signs of disease. Despite technological gains, false alarm rates, or identifying an area of concern when there is not one, persist in medical settings. In recent years, research revealed that half of women experience false positive mammogram results after 10 years of annual screening (Ho et al., 2022). Statistics such as these highlight the prevalence of false alarm rates. The present study investigated ways to leverage the learning process to aid accurate error identification and mitigate false error detection.

Within military settings, personnel must quickly and accurately check equipment (e.g., payloads) for correct set-up. Recognizing errors is important to safety and functional performance but signaling an error when there is not one can hinder efficiency and progress. Ultimately, personnel must rely on their training experience to effectively distinguish between potential errors versus non-meaningful visual differences. Learning from visually complex materials can be challenging, partly because perceivers must consider a host of information when making judgments (Mousavi et al., 1995). Directing learners toward relevant information can aid learning (Mason et al., 2024; Tandoc et al., 2024; Taylor et al., 1999). Salience, understood henceforth as the visual distinctiveness of an item or region, can capture the attention of learners in this manner and support encoding of relevant materials (Britton et al., 1979; Hegarty et al., 2010; LaBerge, 1995; Mason et al., 2024; Pichert & Anderson, 1977; Taylor et al., 1999). Visually salient items can also be distracting, however, resulting in perceivers signaling an error when there is not one, otherwise referred to as a *false alarm.* In the present work, computer renderings of payload models were developed to experimentally investigate modalities to bolster correct error detection and mitigate false alarming in military contexts.

One way to manage error prevalence among operators is by identifying example errors and explaining their underlying problems, affected functions, and potential consequences (Kanse, 2004; Kontogiannis & Malakis, 2009; Rizzo et al., 1994; van der Schaaf, 1995). This approach is supported by elaboration theory which suggests that procedural task performance is best supported by instructional support around why and how something operates (Bradshaw & Anderson, 1982; Reigeluth & Darwazeh, 1982). Based on seminal memory research, memory traces are a record of the level or depth in which stimuli are processed. To this effect, semantically processed materials are expected to be better remembered as compared to those that are not processed in a meaningful way (Bradshaw & Anderson, 1982; Craik & Lockhart, 1972; Craik & Tulving, 1975; Hyde & Jenkins, 1973; Schulman, 1970). One way to enrich encoding within the cognitive domain is through elaboration (Bloom, 1956; Bradshaw & Anderson, 1982; Reigeluth & Darwazeh, 1982). Namely, elaboration theory is a set of blueprints that guide instruction development to meet specific goals. In scenarios where it is imperative for learners to understand why or how something operates, as is the case with the materials used in the present study, emphasizing principles and procedures is advantageous (Reigeluth & Darwazeh, 1982).

Despite the role of elaboration for performance outcomes, analyses of near-miss reports found that error detection is seldom subject to subsequent analysis and explanation of what caused the error (Kontogiannis & Malakis, 2009; van der Schaaf, 1995). Further, to our knowledge, little work directly addresses the beneficial role of elaboration for learning the design and purpose of complex visual information. This question has applied implications, where minor variations across payloads may be misinterpreted as meaningful and wrongfully flagged as an issue in military contexts. To experimentally investigate this scenario and address these gaps in understanding, the present study explicitly assessed whether additional elaboration about errors and differences helped learners more accurately identify errors and correctly reject non-meaningful differences from complex visual materials.

In this study, learners studied a correctly assembled virtual LEGO® model and the same model when it contained errors. In addition to seeing visual examples of errors, learners also received audio explanations about what constituted an error. This combination of visual and audio components aligns with the notion that rich memory traces are supported by rich, multimodal encoding experiences, potentially because they support memory trace access via multiple retrieval cues at test (Bartsch & Oberauer, 2021; Meyerhoff & Huff, 2016). Providing learners with information about the model via simultaneous animation and associated narration also prevented them from having to split visual attention between viewing the model and reading critical information about it. Cross-modality presentation has been found effective with multimedia learning (Mayer, 1989; Mayer & Anderson, 1991, 1992; Mayer & Gallini, 1990; Mousavi et al., 1995). In accordance with elaboration theory tenants, the elaboration provided followed a general-to-detailed sequence about the model components (Reigeluth & Darwazeh, 1982). Then, they were tested on their ability to detect errors within the models. We extended previous findings by explicitly assessing whether explanation of what comprises an error and additional elaboration about potential consequences of the error aids accurate error detection and mitigates inaccurate false alarming over and above explanation alone.

Although much of the research on error management focuses on strategies to promote error detection, the aforementioned issue of “*false alarms*”, or falsely identifying errors that do not exist, remains important. Studies on false alarming are largely concerned with integrating automated systems to reduce false alarm rates. Such approaches are less feasible in certain contexts, such as with the soldier scenario provided earlier. Additionally, little work has examined the cognitive processes underlying false alarming during complex visual error detection tasks. Some findings suggest misleading cues that appear contrary to one’s preexisting knowledge structure for learned materials may inhibit error detection (Cohen et al., 1996; Kontogiannis & Malakis, 2009). Alternative sources suggest that when presented with conflicting cues, effective operators strategically elaborate on a story to explain the cue rather than simply discarding it (Cohen et al., 1996). In sum, it remains unclear how the initial presentation of complex materials can be leveraged to mitigate false alarming during later visual error detection protocols. In the present work, we strategically employed an instructional manipulation to assess whether elaborative information at learning can aid performance accuracy at test. In collective consideration of findings from the elaborative encoding and multimedia literature broadly, we anticipated that guiding learners to visual differences in models and providing them with simultaneous auditory elaborations about why differences were not errors might concurrently enrich the memory trace for learned materials and prepare learners to refer to relevant visual information when making error judgments at test.

The present study extended findings from prior work by examining the role of additional elaboration for (1) error detection and (2) false alarm rejections. Learners studied errors and differences from virtual LEGO® models. Here, *differences* were properties within the models that were visually dissimilar from the base model (Rensink, 2002) and did not pose an underlying problem, affect functions, or result in potential negative consequences. Despite their lack of importance for model integrity, differences in the present study, such as a colorful battery box, could prove salient and involuntarily capture learners’ attention at test (Rensink, 2002). Such capture might impair one’s ability to accurately reject differences as non-errors, thereby signaling a false alarm.

Additionally, we argue that the presence of differences mirrors ecologically relevant scenarios, where material features may vary across manufacturers or time periods. Such differences could function as cues that disturb recognition patterns, warrant unnecessary explanation from the operator, and wrongfully signal an error. We experimentally tested this claim in the present study by assessing whether learners who were provided with explicit elaboration about differences (namely why they were not an error) better rejected them as non-errors as compared to those who were simply told that they were not an error but did not receive additional elaboration about *why* the difference did not constitute an error.

## Experiment 1

In Experiment 1, learners first studied a correct virtual LEGO® model. Then, they studied models containing three categories of errors and two categories of differences before their ability to identify correct models, detect errors, and reject differences as non-errors was tested. We hypothesized that learners who received additional elaboration about errors and differences would more accurately identify errors and correctly reject differences at test.

### Method

#### Participants

Eighty-five participants ages 18–27 were recruited from Tufts University through SONA and were compensated with class credit. Most participants identified as White (61%) or Asian (31%) females (62%). They completed the experiment in the laboratory on a laptop or desktop device.

Due to the novel stimuli, experimental paradigm, combined theoretical bases, and statistical analysis methods employed in this study, we determined the desired sample size through holistic consideration of previous work from the multimedia and instructional science literature broadly (Bradshaw & Anderson, 1982; Corral & Carpenter, 2020; de Koning et al., 2010; García Rodicio et al., 2013; Mousavi et al., 1995). These studies utilized a variety of designs and ranged in participants numbers from 19 on the low end and 341 at the high end. Given the modest complexity of our research design and robustness of our planned multilevel modeling analysis approach, we aimed to analyze data from 80 participants in each experiment.

#### Design

The experiment used a 2 (condition: basic, elaboration) × 3 (trial type: correct, difference, error) mixed design. Half of the participants received basic information about errors and differences (basic condition) and half received additional elaboration about errors and differences (elaboration condition).

### Materials

#### Virtual cargo bag model

**Fig. 1:**
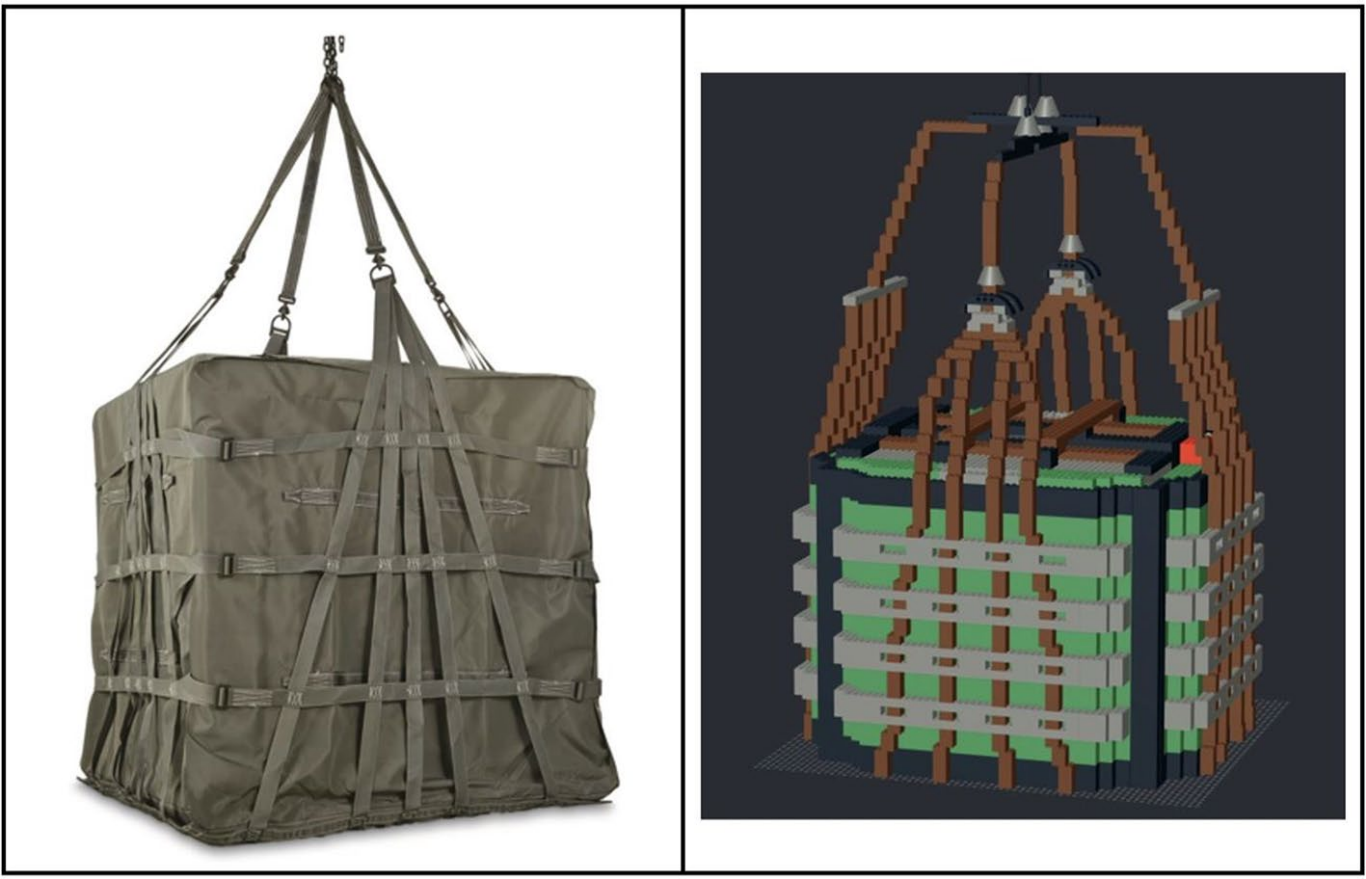
The A22 (left) is used as a cargo bag for aerial delivery to air drop supplies to U.S. Armed Forces. The P-A22 (right) was modeled loosely after the A22 for experimental purposes

The virtual P-A22 LEGO® model in Experiment 1 was developed using BrickLink Studio software *(bricklink.com)* and was loosely modeled after the A22 cargo bag utilized in military contexts. A comparison of the A22 bag and the P-A22 stimulus model is provided in Fig. 1.

#### Learning materials

**Fig. 2:**
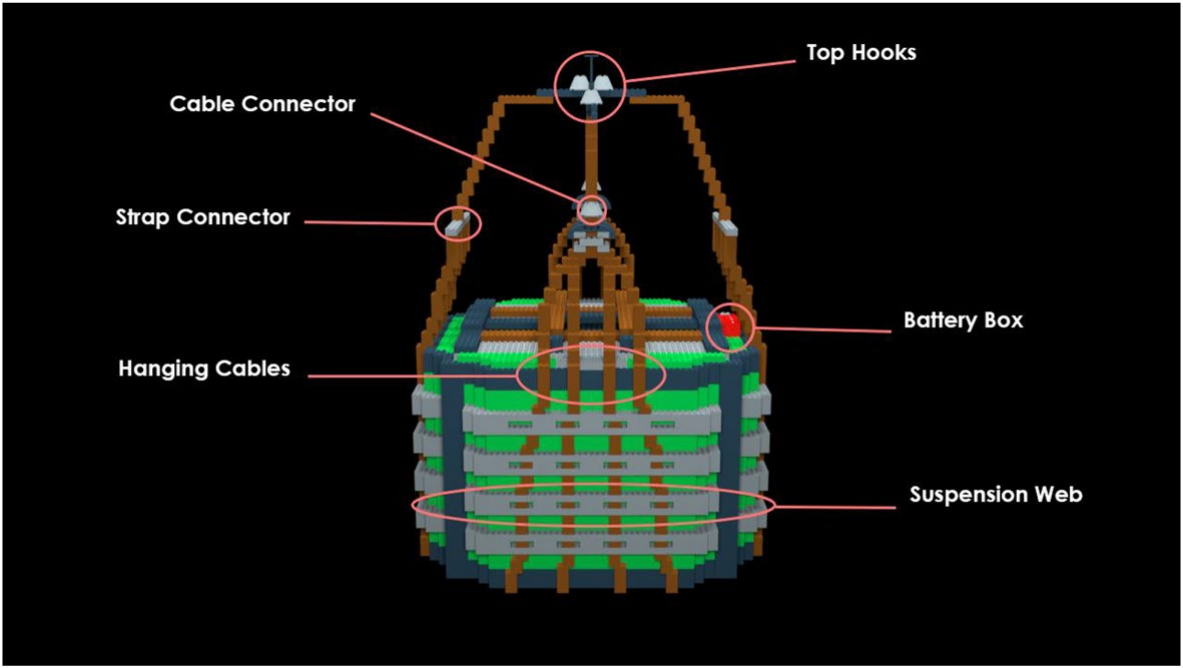
Front-facing annotated pre-learning photograph of the P-A22 bag model with bag parts labeled

**Fig. 3:**
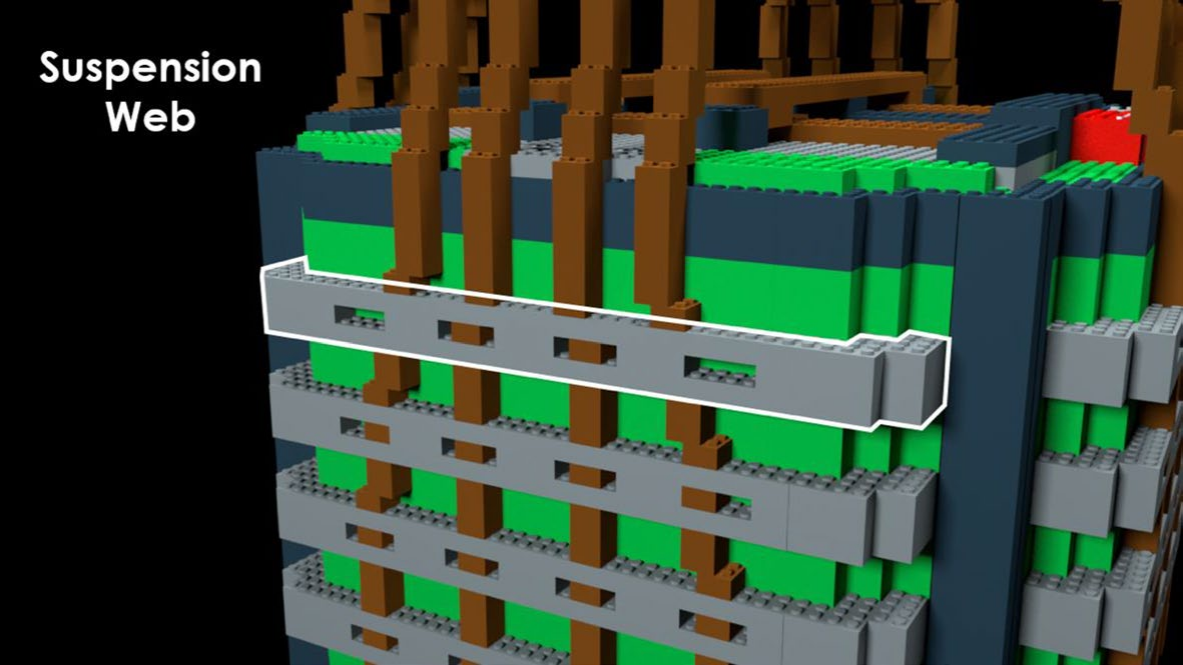
Zoomed-in, annotated pre-learning photograph with “suspension web” part highlighted

*Pre-learning photos*. There were 12 annotated photographs of a correctly-rigged bag. Each photograph highlighted distinct bag components. The components were designed such that they could contain an error or difference later in the study. Two of the 12 photographs for the P-A22 bag model showed a wide view of the full bag with part names labeled. One photograph showed the front-view of the bag (Fig. 2) and the other showed a top-view. The remaining ten photographs each featured a zoomed-in view of one specific bag part (Fig. 3). The various photographs featured different angles of the bag in a two-dimensional view.

*Pre-learning videos*. In addition to the pre-learning images, there was one pre-learning video. The video showed the correctly-rigged bag rotating to highlight the different sides and provide a three-dimensional perspective of the bag. This video paralleled the learning videos explained later and thereby provided a preview of the learning video format.

**Fig. 4:**
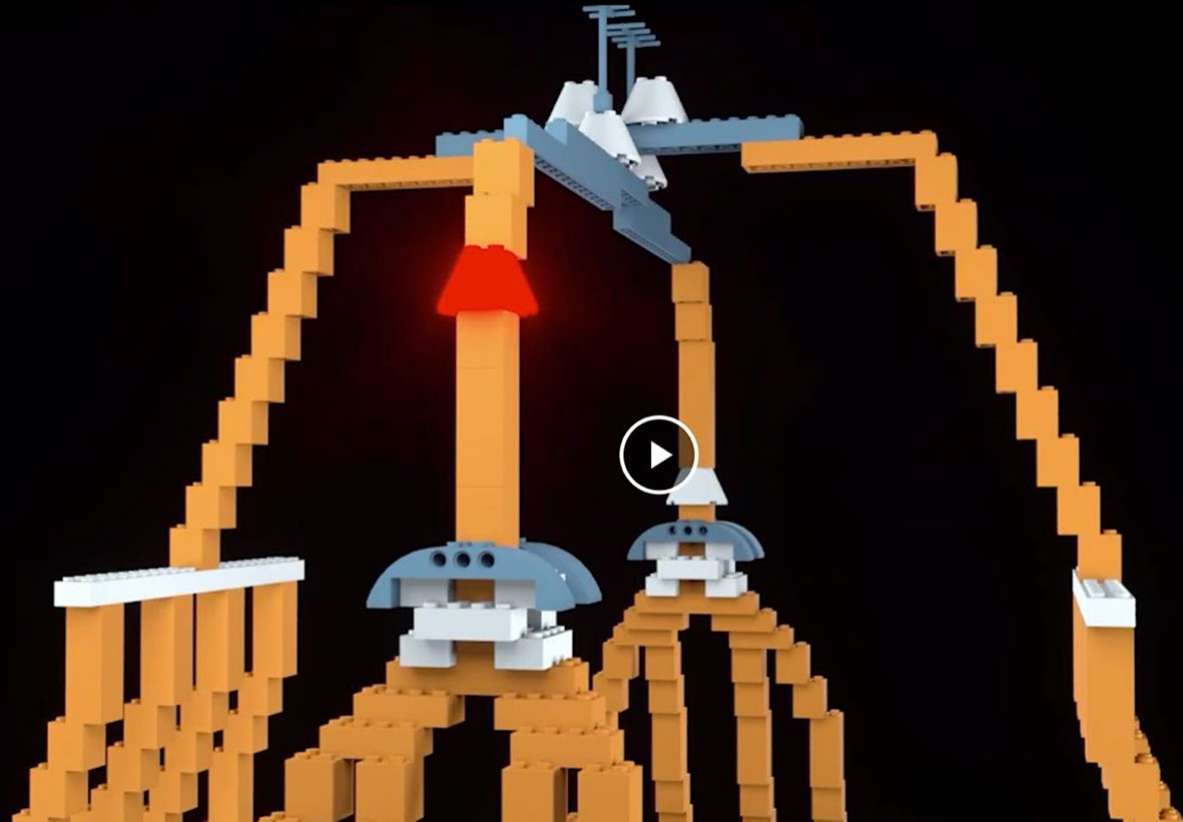
Example of a learning video zoomed-in to highlight a location error. The highlighted part was shown glowing red and flashed to indicate the error

*Learning videos*. There were 14 learning videos. Each video corresponded to one of 10 errors or four differences. The format of these videos was similar to the pre-learning videos in that they showed a full rotation of the entire bag model to give a three-dimensional perspective. After the full rotation, the video zoomed in on a portion of the bag that included an error or difference highlighted with a flashing translucent glow that signified the focus of the learning video (Fig. 4).

The learning videos demonstrated how the bag could be correctly or incorrectly rigged**.** Videos featured both errors and non-error differences. The bag could contain three categories of errors: *location, omission*, and *orientation*. Location errors were incorrect placements of a bag part, omission errors involved a missing bag part, and orientation errors were slight, incorrect variations in the position of a part. There were four examples of location errors, four examples of omission errors, and two examples of orientation errors, totaling to ten error examples.

In contrast to errors, differences were variations in how a bag part looked compared to the correctly-rigged model, but they had no effect on the bag’s function. All participants were explicitly told that differences were not errors. In alignment with typical payload conditions, real-world continuous visual search tasks (Chan & Chan, 2022), and stimulus constraints broadly, we employed a smaller proportion of difference targets as compared to correct or error targets. There were two categories of differences: color and addition. Color differences showed a bag part in a different color, and addition differences contained extra parts than were displayed on the correctly-rigged model. For each bag, there were two examples of color differences and two examples of addition differences, totaling to four difference examples. A breakdown of the categories, descriptions, and number of instances that they occurred is provided in Table 1.

**Table 1:** Error and difference categories, descriptions, and number of instances that they occurred

Error category	Category description	No. of instances
Location	Incorrect placement of a bag part	4
Omission	Missing a bag part	4
Orientation	Incorrect positioning of a bag part	2

Difference category	Category description	No. of instances
Color	A different color of bag part	2
Addition	Extra bag part	2

*Learning video voiceover recordings*. Each learning video contained a corresponding voice narration that explained the error or difference highlighted in the video. The amount of explanation that the voiceover gave differed between the basic and elaboration conditions. Each voiceover included information about the error categorization and error identification. For the elaboration condition, a third piece of information was also provided. For participants in the basic condition, the voiceover only explained the first two explanatory aspects (error categorization and error identification). The length of videos was matched across conditions despite differences in the amount of information included in the voiceover. An outline of the voiceover components is provided (Table 2).

**Table 2:** P-A22 voiceover learning audio materials

Error category	Error identification	Error elaboration (explanation + consequence)
Location	The cable connector near the top of the bag is located too high	If the cable connectors are located too high, the straps cannot sway in the wind during flight. This can cause the straps to break
Location	Part of the top hook is misplaced	If part of the top hook is misplaced, the bag cannot connect correctly to the airplane. This can cause the bag to fall during flight
Location	The cable connectors are misplaced onto the adjacent hanging straps	If the cable connectors are misplaced, the weight of the bag is unbalanced. This can cause the bag to shift during flight
Location	The center straps on the top of the bag are wider apart	If the center straps on top of the bag are wider apart, the straps below them can become loose in transit. This can cause them to lift and disconnect from the bag
Omission	The battery box is missing	If the battery box is missing, there is no way to track the location of the bag. This can result in the bag and its contents becoming lost
Omission	One of the side grab handles is missing	If a grab handle is missing, it makes it difficult to move the bag. This can prevent the bag from being transported when it is on the ground
Omission	One of the suspension webs surrounding the bag is missing	If a suspension web is missing, the cables can fall away from the bag. This can weaken the strength of the cables
Omission	The center, inferior strap is missing	If the center, inferior strap is missing, there is too much tension placed on the surrounding straps. This can cause surrounding straps to break
Orientation	The hanging cables are twisted	If the hanging cables are twisted, the bag can begin to spin during flight. This can create dangerous flight conditions
Orientation	The cable joints are turned	If the cable joints are turned, the cables can disconnect. This can place excess weight on other straps

Difference category	Difference identification	Difference elaboration (explanation + consequence)
Color	The battery box is a different color	Even though the battery box is a different color, the color only reflects the manufacturer. This is not an error
Color	Part of the hanging cable is a different color	Even though part of the hanging cable is a different color, this only reflects which team attached the cable. This is not an error
Addition	There are additional center straps on top of the bag	Even though there are additional center straps on top of the bag, this will add to the security of the bag. This is not an error
Addition	There are additional suspension webs	Even through there are additional suspension webs, this only means that the bag has been transported multiple times. This is not an error

#### Error detection test materials

**Fig. 5:**
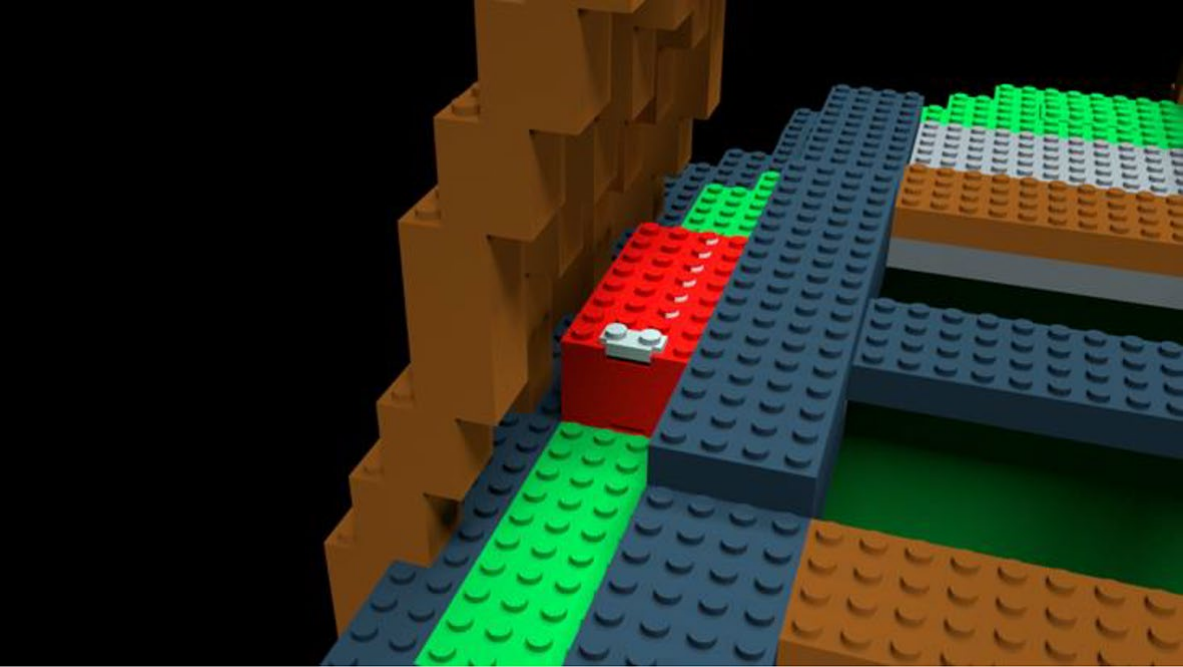
Example error detection test photograph of a correct model that does not contain an error

**Fig. 6:**
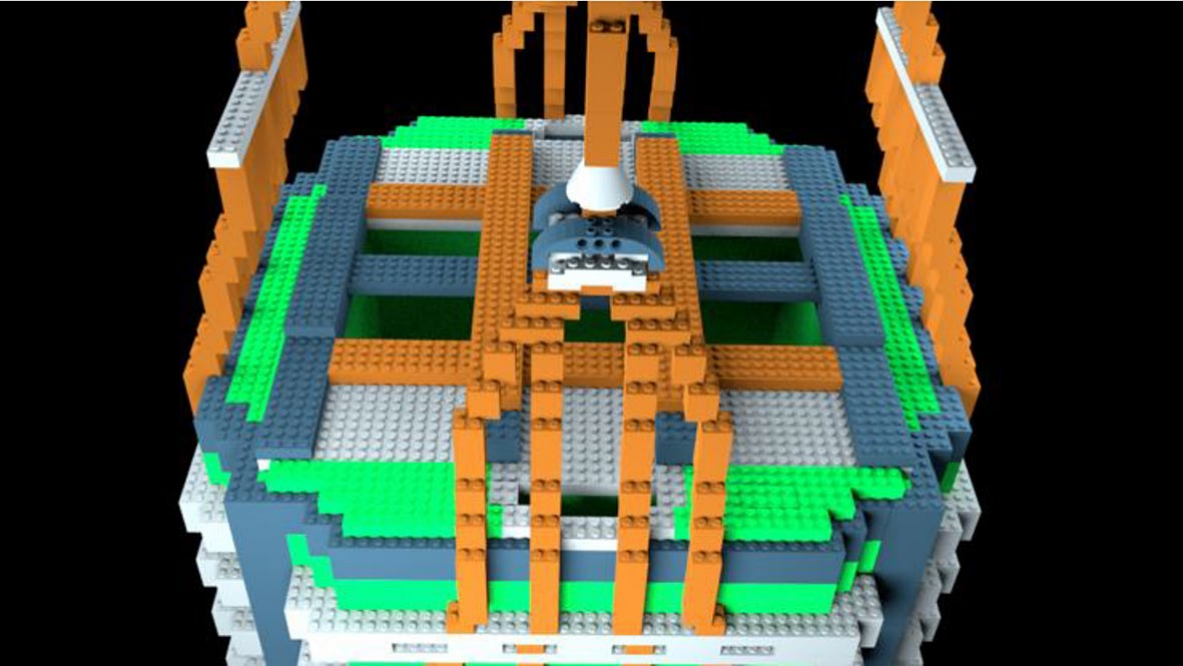
Example error detection test photograph of an omission error (*Note:* the battery box is missing)

**Fig. 7:**
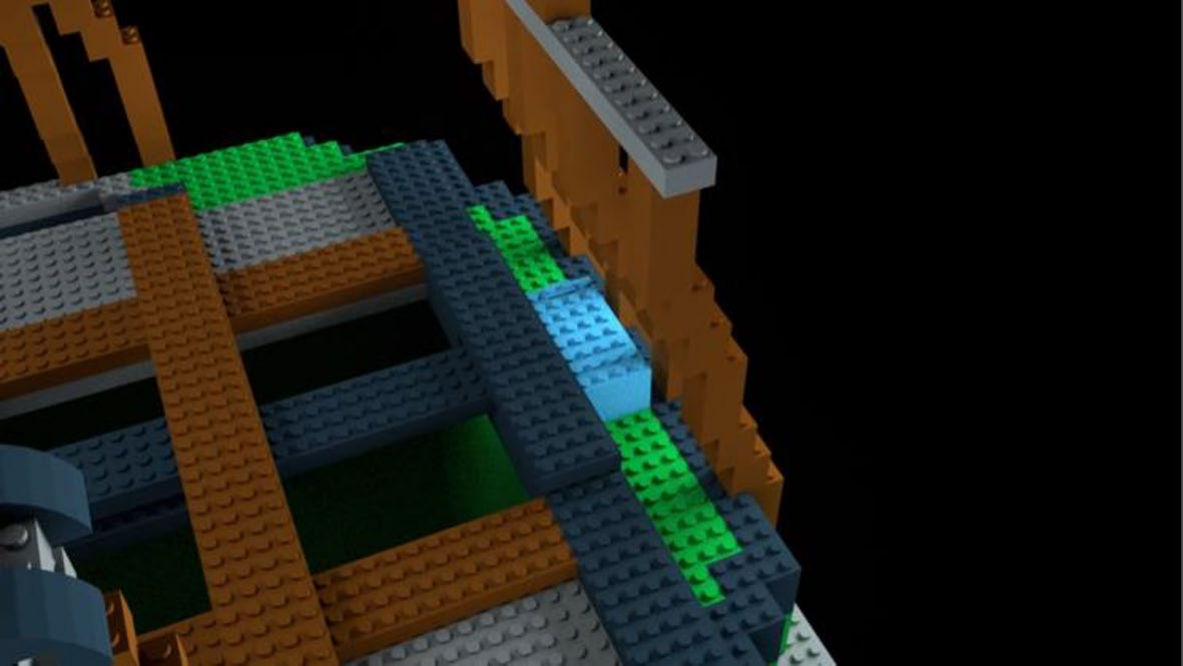
Example error detection test photograph of a color difference (*Note:* the battery box is a different color)

For the purpose of an error detection test, a set of 24 photographs was developed. The set consisted of ten error photographs, ten correct photographs, and four difference photographs. The photographs were the same as those shown during pre-learning, except now they did not contain highlights or labels. Example error detection test photographs of a (1) correct model, (2) omission error, and (3) color difference are provided in Figs. 5, 6, and 7.

#### Retention interval

A black and white drawing containing a list of hidden items was used during a two-minute retention interval. The hidden picture printable for adults were pulled from the internet (https://www.printablee.com).

#### Questionnaire

There was a demographics questionnaire included at the end of the study that asked about participant gender, ethnicity, highest degree completed, and age.

### Procedure

All participants completed the experiment in the laboratory using the PsychoPy software desktop application *(PsychoPy.org).* The experiment consisted of five main stages: Pre-Learning, Learning, Retention Interval, Test, and Questionnaire.

#### Scenario

At the start of the experiment, participants were told that their “assignment is to learn how to check a bag to ensure that it is correctly prepared for flight.” Further, they were instructed to “Imagine that this bag will connect to an airplane and will eventually be dropped from the plane” and they were made aware that they would learn about potential errors that could occur when the bag was prepared incorrectly and potential differences that look different but are not errors. Then, the pre-learning phase began.

#### Pre-learning phase

The pre-learning phase allowed participants to get accustomed to the materials and parts of the bag model. Participants first viewed the front and top photographs of the bag containing labeled part names. Then, the program zoomed in to allow learners to study each labeled part more closely for 5 s. Next, they viewed a video of the bag rotating to view all sides of the bag in a three-dimensional perspective before beginning the learning phase.

#### Learning phase

At the start of the learning phase, participants were told that it was time to learn about potential errors and differences that could occur during bag preparation. They were also reminded to imagine that the bag would be dropped from an airplane, and it was important that there were no errors.

Participants viewed a series of 14 videos to learn about the ten errors and four differences that could appear on the bag. Each video was 35 s long. While watching, the participants listened to a voiceover explaining the information relevant to the error or difference. The content for the voiceovers depended on the elaboration condition to which the participant was assigned. The basic condition heard voiceovers containing the error categorization and error identification, while the elaboration complex condition heard voiceovers containing all three explanatory aspects: error categorization, error identification, and error elaboration. After each video, participants saw a fixation cross before moving on to the next video. The order of videos was randomized.

#### Retention interval

Between the learning and test phases, participants completed the hidden pictures retention interval. After viewing the image and looking for the hidden items listed at the bottom of the page for two minutes, they were automatically forwarded to a screen that prompted them to report the number of items they found. Then, they moved on to the test phase.

#### Error detection test

Participants completed an error detection test to measure their ability to identify correct models, detect errors, and correctly reject differences as non-errors. They were told that they would view the same photographs that they saw during learning and their task was to press the ‘y’ key if they detected an error in the bag photograph and the ‘n’ key if they did not detect an error. They viewed and responded to a series of 24 photographs (10 correct, 10 errors, and four differences). The presentation of test photographs was randomized, and participants saw a fixation cross between each test trial.

#### Questionnaire

After completing the test, participants filled out the demographics questionnaire.

## Results

We used RStudio Version 2023.12.1+402 (2023.12.1+402) to complete all analyses. We estimated generalized linear mixed models with 1) the binary outcome variable, detection accuracy, predicted by condition (basic, elaboration), a binary between-subjects predictor, and trial type (correct, difference, error), a within-subjects predictor.

Linear mixed models are beneficial because they allow one to account for variations in performance attributable to random effects of item and participant. To evaluate the variation in detection accuracy by stimulus and participant, we estimated a series of empty models. We evaluated the ICC and conducted likelihood ratio tests after estimating each subsequent model to see whether addition of the random effect in question decreased the model deviance.

The first empty model included a random effect for the intercept for stimulus and revealed that stimulus accounted for 17% of the variation in detection accuracy. The second empty model included a random effect for the intercept for participant, revealing that 19% of the variation in accuracy was accounted for by this random effect. Random intercepts for stimulus and participant were therefore retained in the model predicting detection accuracy.

We proceeded by estimating our hypothesized detection accuracy model. We added a fixed effect of condition and its interaction with trial type to test whether learners in the elaboration condition exhibited greater detection accuracy and whether this pattern varied across trial type. This model (E1ME, Table 3) was a better fit for the data than the null model based on AIC and log likelihood value comparisons (*χ*^2^ (5) = 100.4, *p* < .001).

**Table 3:** Model formulas and associated AIC values

Model	Model formula	AIC
E1ME	Accuracy ~ 1 + (1|Participant) + (1|Stimulus)	1494.7
E1M1	Accuracy ~ Condition*Trial Type + (1|Participant) + (1|Stimulus)	1404.4

**Fig. 8:**
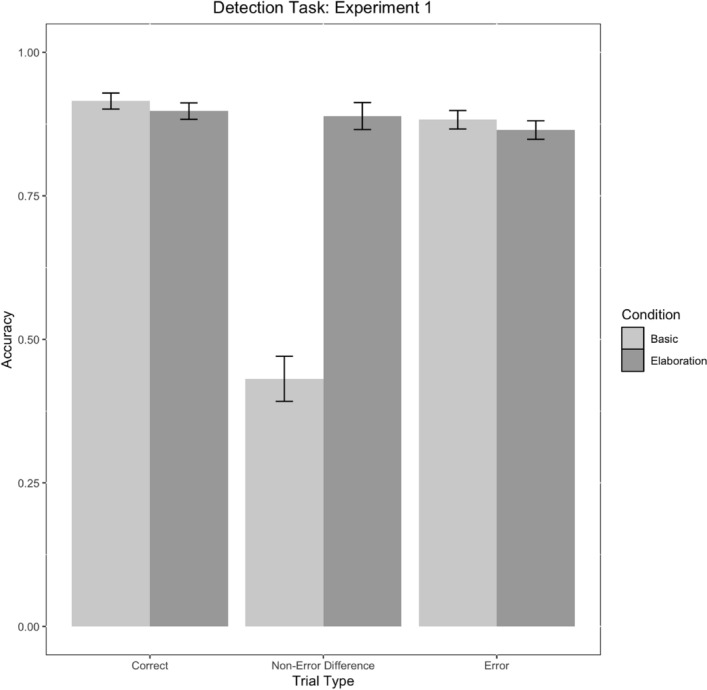
Detection task accuracy by condition and trial type in Experiment 1. Error bars represent standard errors

We observed main effects of condition, trial type, and a condition*trial type interaction (Table 4). Critically, participants in the elaboration condition were less likely to false alarm to differences as compared to the basic condition (Fig. 8).

**Table 4:** Parameter estimates for model E1M1

Fixed effects	Estimate	*SE*	*No. Obs*	*Z*	*p*	95% CI [LL, UL]
*Parameter*						
Intercept	2.11	0.21	2039	10.44	< .001***	[5.54, 12.21]
Condition	− 0.45	0.14	2039	− 3.28	< .01**	[0.49, 0.83]
Trial Type: Correct versus Other	0.32	0.10	2039	3.16	< .01**	[1.12, 1.69]
Trial Type: Difference versus Error	− 0.63	0.21	2039	− 3.05	< .01**	[0.36, 0.80]
Condition*Trial Type: Correct versus Other	0.25	0.05	2039	4.57	< .001***	[1.15, 1.42]
Condition*Trial Type: Difference versus Error	− 0.73	0.11	2039	− 7.45	< .001***	[0.40, 0.58]

Condition Levels: 0 = Elaboration, 1 = Basic. ‘Other’ = Difference and Error trial types combined. No. Obs = Number of observations

***p < .001, **p < .01, *p < .05

## Discussion

In Experiment 1, we found that learners who received elaborations more accurately rejected non-error differences at test. In contrast, they did not more accurately detect errors or identify correct models, though we note that accuracy across correct and error models approached ceiling across both conditions. The finding that learners who received elaboration better identified non-error differences aligned with our prediction that receiving additional explanation about what constitutes errors and characterizes differences could aid error detection outcomes. Specifically, learners who received elaboration were more successful at correctly distinguishing when a difference was not an error. Learners in the basic condition, who did not receive additional elaboration about errors, performed *below* chance at detecting non-error differences. This observation may suggest that participants in the basic condition viewed any difference as a meaningful error. One possibility is that they effectively learned how to identify a correct model but were less successful at accurately distinguishing between non-error differences versus errors, resulting in a response bias toward errors in the absence of sufficient learning.

Taking together, these findings are especially relevant for informing how false alarm detection can be mitigated. In addition to training operators on how to detect errors, our findings suggest that it is similarly critical to provide explanation for complex visual display elements that appear different but are not errors. Such training may alleviate tendencies to falsely characterize differences as errors. In Experiment 2, we (1) test these findings again using a different LEGO® model, and (2) test whether elaboration on one model extends to support error detection on a second model.

## Experiment 2

In Experiment 2, learners again studied three categories of errors and two categories of differences from a complex virtual LEGO® model and their ability to correctly detect errors and reject differences as non-errors was tested. Now, we (1) replicated findings from Experiment 1 with a new virtual LEGO® model, and (2) extended findings by having participants learn and be tested on a second model. Given the results of Experiment 1, we hypothesized that learners who received additional elaboration about errors and differences on the first bag would more accurately reject differences as non-errors on both bags (i.e., we predicted that a transfer effect would be observed).

### Participants

Ninety-eight participants ages 16–25 were recruited from Tufts University through SONA and were compensated with class credit. Participants under the age of 18 received parental consent to participate in research. Most participants identified as White (57%) or Asian (28%) females (65%). They completed the experiment in the laboratory on a laptop or desktop device.

### Design

The experiment used a 2 (condition: basic, elaboration) × 3 (trial type: correct, difference, error) mixed design. In Part 1 of the experiment, half of the participants received basic information about errors and differences (basic condition) and half received additional elaboration about errors and differences (elaboration condition) while learning the first bag. While learning the second bag in Part 2, all participants received basic information, but no one received additional elaboration about errors and differences.

### Materials: Experiment Part 1 (D-A22)

#### Virtual Cargo bag models

**Fig. 9:**
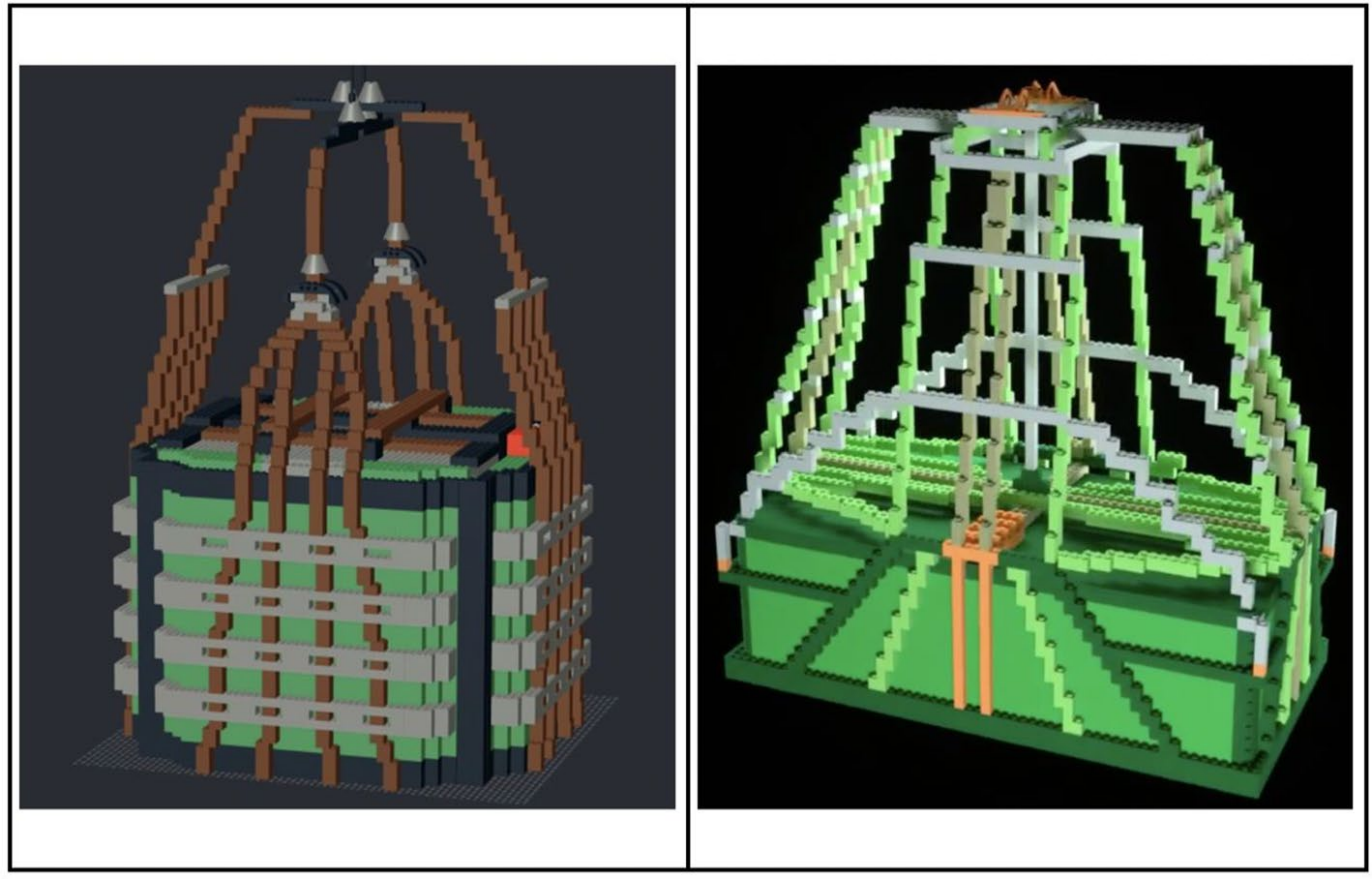
The P-A22 (left) and D-A22 (right) models used in Experiment 1 and Part 1 of Experiment 2, respectively

In Part 1 of the present experiment, a new bag model identified as the D-A22 was used. The purpose of using a new model in Part 1 was to test whether findings from Experiment 1 could be replicated with different materials. A comparison of the P-A22 and D-A22 stimulus models is provided in Fig. 9.

#### Learning materials

**Fig. 10:**
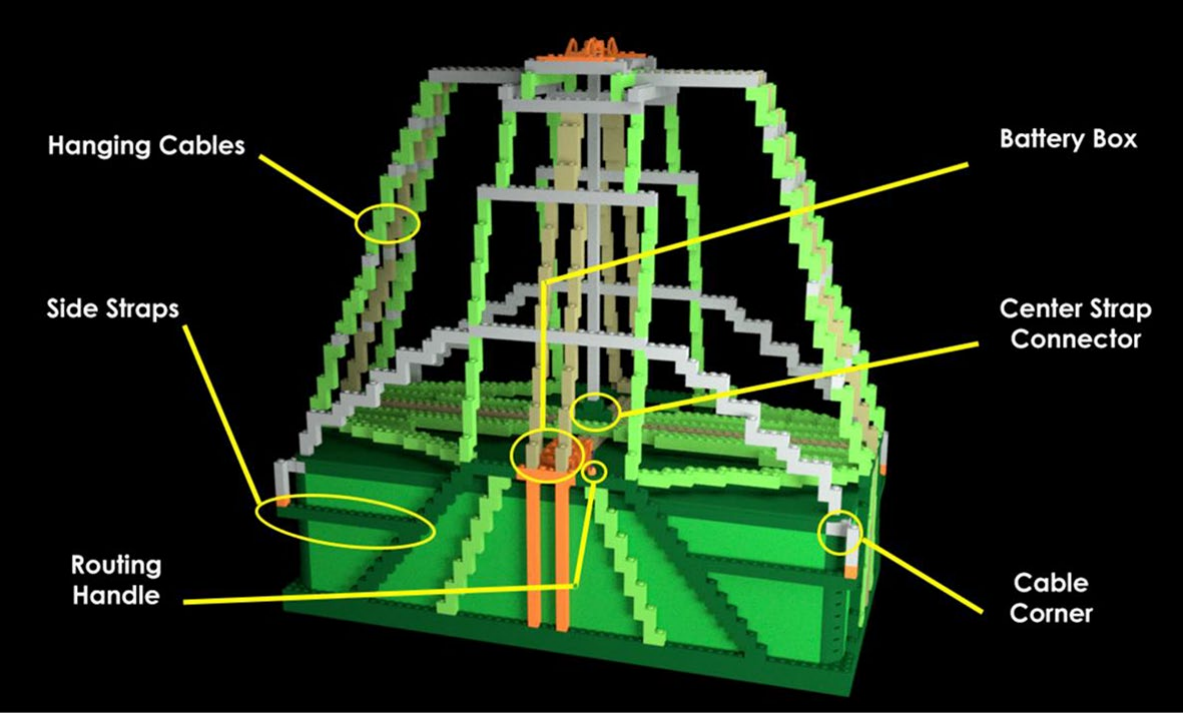
Left-facing annotated pre-learning photograph of the D-A22 bag model with bag parts labeled

**Fig. 11:**
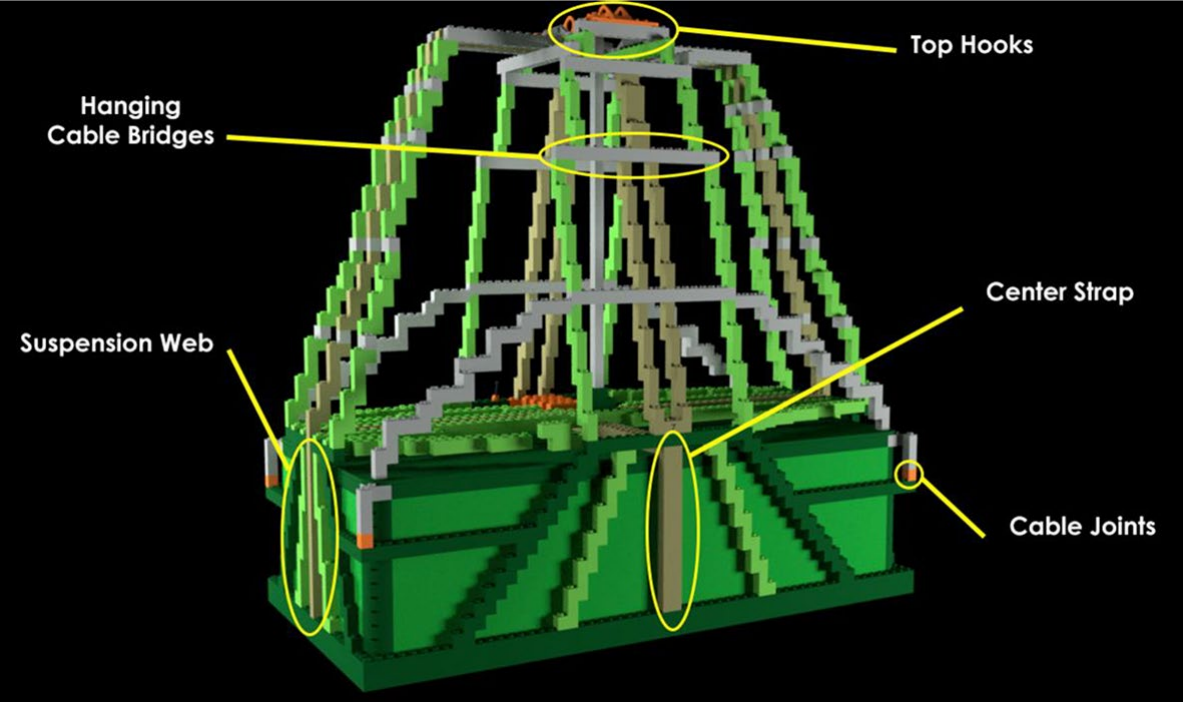
Right-facing annotated pre-learning photograph of the D-A22 bag model with bag parts labeled

**Fig. 12:**
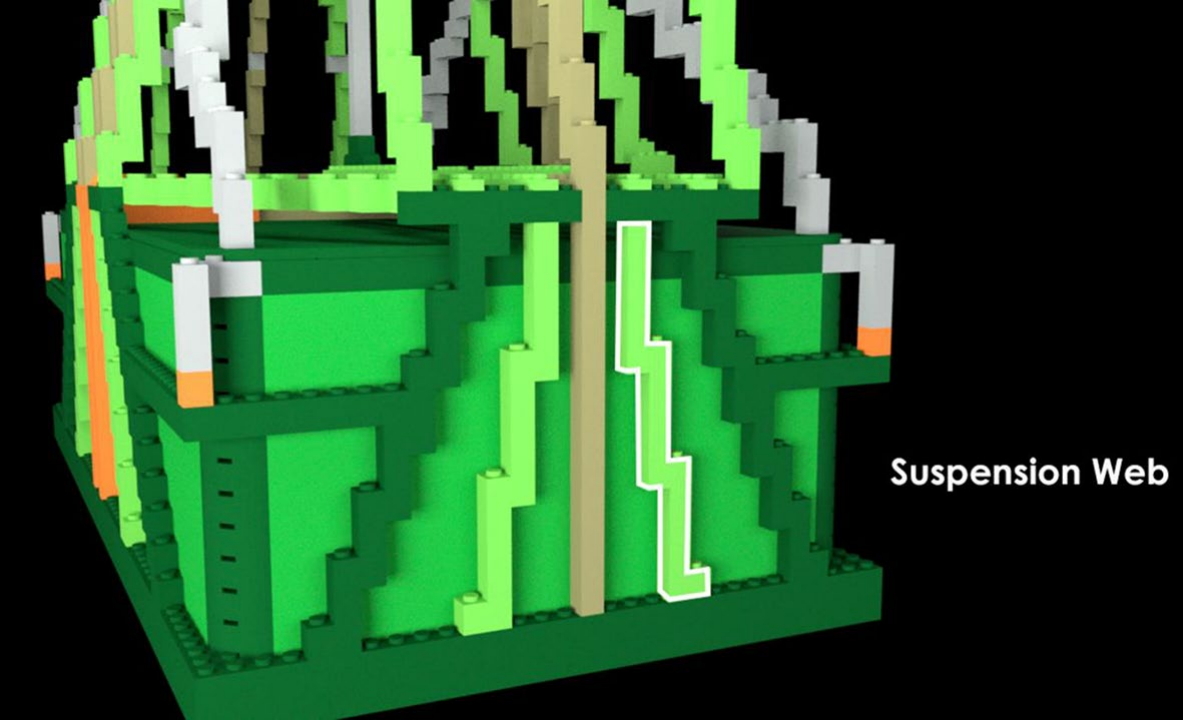
Zoomed-in, annotated pre-learning photograph with “suspension web” part highlighted in Experiment 2, Part 1

*Pre-Learning Photos*. Just as in Experiment 1, there were 12 annotated photographs of the correctly-rigged bag (now the D-A22). Two of the 12 photographs showed a left (Fig. 10) and right-side (Fig. 11) view of the bag model with the part names labeled. The remaining ten photographs were each zoomed-in views of a specific bag part (Fig. 12).

*Pre-Learning Videos*. The pre-learning video for Part 1 was the modeled after the one used in Experiment 1, except now it featured the D-A22 bag model.

**Fig. 13:**
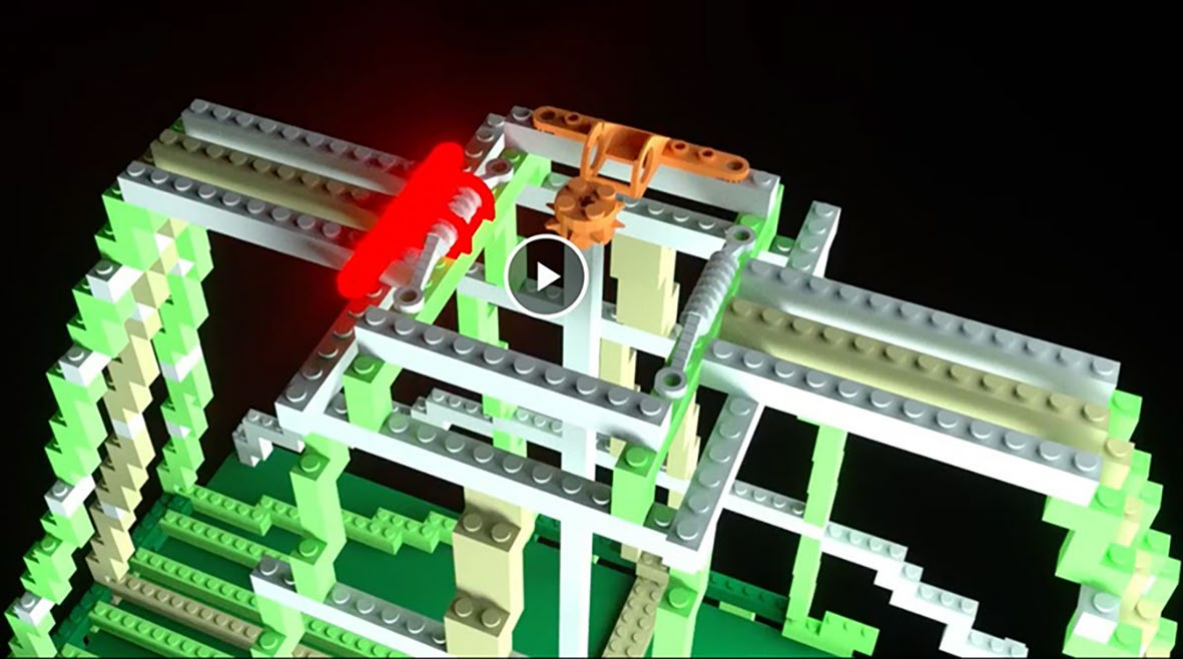
Example of a learning video zoomed-in to highlight a location error in Experiment 2, Part 1. The highlighted part was shown glowing red and flashed to indicate the error

*Learning Videos*. There were again 14 learning videos corresponding to one of 10 errors or four differences. The videos modeled those used in Experiment 1 (Fig. 13).

*Learning Video Voiceover Recordings*. New voiceover recordings were developed for Experiment 2, Part 1. The terminology used and errors employed are similar to those used in Experiment 1. An outline of the voiceover components is provided (Table 5).

**Table 5:** D-A22 voiceover learning audio materials

Error category	Error identification	Error elaboration (explanation + consequence)
Location	The center strap is not located in the center of the bag	If the center strap is not located in the center of the bag, the weight of the bag is not distributed evenly. This can cause the bag to shift during flight
Location	The center strap connector is not located in the center of the bag	If the center strap connector is not located in the center of the bag, the center strap can lift from the bag. This can cause straps to disconnect
Location	The routing handle is located opposite from the battery box instead of next to it	If the routing handle is not located next to the battery box, they cannot sync electronically. This can prevent the bag from being tracked
Location	The top hook is adjacent to the other one instead of across from it	If the top hooks are not across from one another, the weight of the bag is not evenly distributed. This can cause the bag to fall during flight
Omission	One of the cable joints is missing	If a cable joint is missing, the cable can slip out and disconnect. This can place excess weight on other cables
Omission	One of the hanging cables is missing	If a hanging cable is missing, there is too much tension placed on the surrounding straps. This can cause surrounding straps to break
Omission	The battery box is missing	If the battery box is missing, there is no way to track the location of the bag. This can result in the bag and its contents becoming lost
Omission	One of the top hooks is missing	If a top hook is missing, the bag cannot connect to the airplane. This prevents the bag from being air lifted to locations
Orientation	One of the cable corners is not secured	If a cable corner is not secured, the cable can fall away from the bag. This can weaken the strength of the cable
Orientation	The hanging cable is tangled	If a hanging cable is tangled, the bag can begin to spin during flight. This can create dangerous flight conditions

Difference category	Difference identification	Difference elaboration (explanation + consequence)
Color	The battery box is a different color	Even though the battery box is a different color, the color only reflects the manufacturer. This is not an error
Color	Part of the hanging cable is a different color	Even though the suspension web is a different color, this only reflects which team attached the web. This is not an error
Addition	There are additional center straps on top of the bag	Even though there is an additional side strap, this will add to the security of the bag. This is not an error
Addition	There are additional suspension webs	Even through there is an additional hanging cable bridge, this only means that the bag has been transported multiple times. This is not an error

#### Error detection test materials

**Fig. 14:**
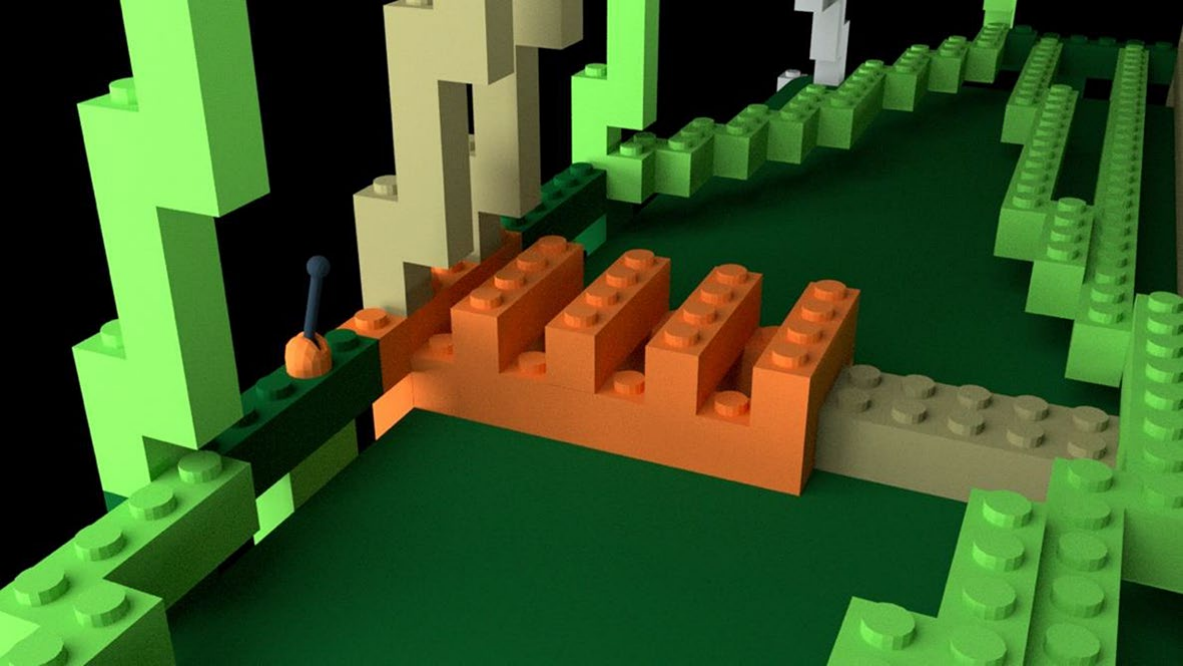
Example error detection test photograph of a correct model that does not contain an error (Experiment 2, Part 1)

**Fig. 15:**
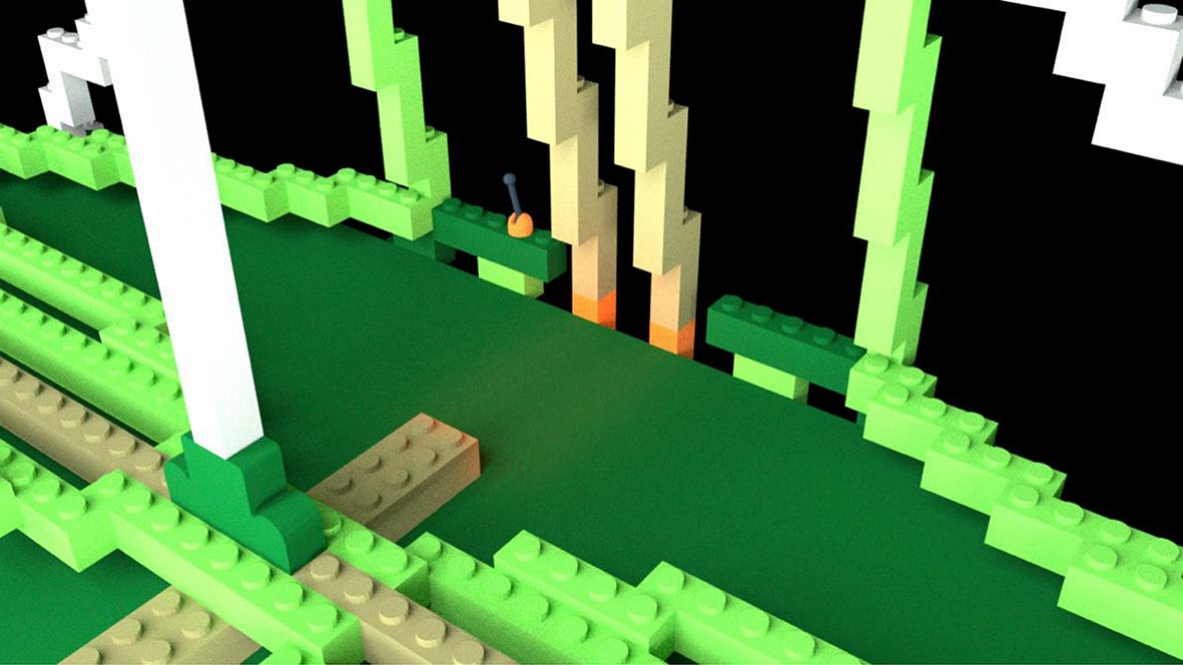
Example error detection test photograph of an omission error (Experiment 2, Part 1) (*Note:* the battery box is missing)

**Fig. 16:**
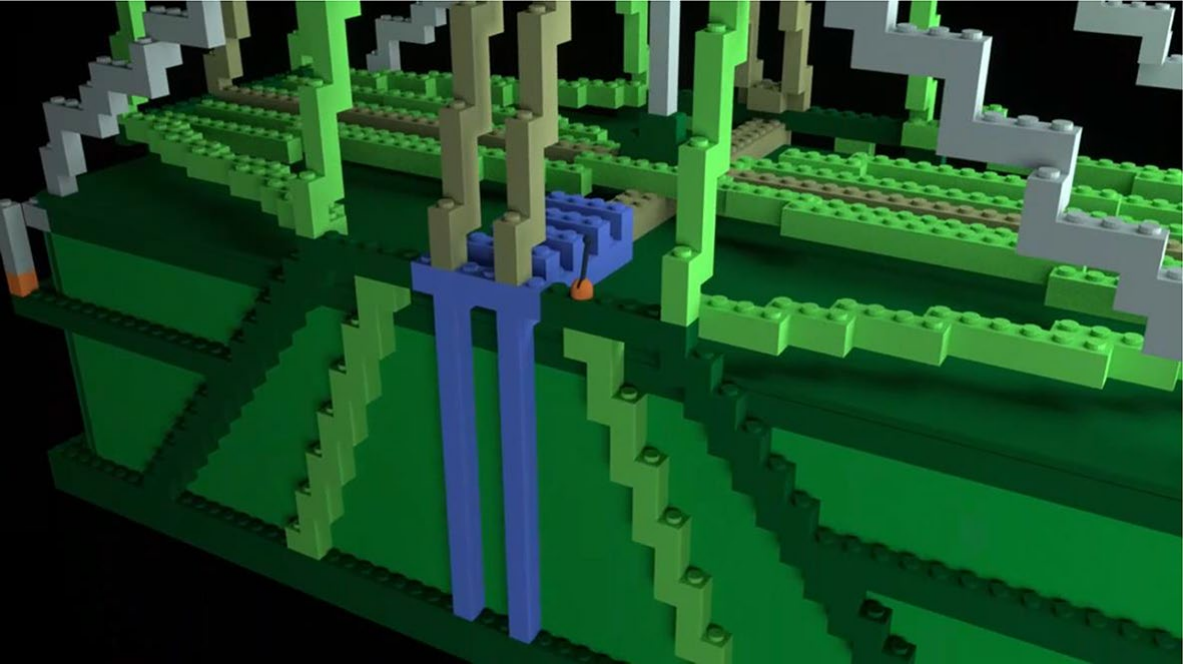
Example error detection test photograph of a color difference (Experiment 2, Part 1) (*Note:* the battery box is a different color)

There were again 24 photographs, 10 of which contained errors and 10 of which did not contain errors, and 4 of which contained differences. Example error detection test photographs of a (1) correct model, (2) omission error, and (3) color difference are provided in Figs. 14, 15, and 16.

#### Retention Interval

The same retention interval from Experiment 1 was used.

#### Questionnaire

The same questionnaire from Experiment 1 was used.

### Materials: Experiment Part 2 (P-A22)

For Part 2 of the experiment, all materials were the same as in Experiment 1. Now, we only used the basic voiceover for both conditions. A second hidden pictures retention interval was also used.

### Procedure

All participants again completed the experiment in the laboratory. Now, the experiment was broken into Part 1 and Part 2. In Part 1, participants completed a pre-learning phase before learning, retention interval, and testing on the D-A22. They then repeated this process with the P-A22 before completing the demographics questionnaire. The scenario was the same as in Experiment 1, except now learners studied and were tested on two bags instead of just 1, and no one received additional elaboration while learning in Part 2. A retention interval was placed between the learning and test phases in both Part 1 and Part 2. After learning and being tested on both bags, learners completed the questionnaire.

## Results

We again estimated multilevel models to test whether learners in the elaboration condition better detected errors at test, beginning with Part 1 of the experiment. The first empty model included a random effect for the intercept for stimulus and revealed that stimulus accounted for 36% of the variation in detection accuracy. The second empty model included a random effect for the intercept for participant, revealing that 16% of the variation in accuracy was accounted for by this random effect. Random intercepts for stimulus and participant were therefore retained in the model predicting detection accuracy.

We proceeded by estimating our hypothesized detection accuracy model. We added a fixed effect of condition and its interaction with trial type to test whether learners in the elaboration condition exhibited greater detection accuracy and whether this pattern varied across trial type. This model (E2M1, Table 6) was a better fit for the data than the null model based on AIC and log likelihood value comparisons (*χ*^2^ (5) = 179.3, *p* < .001).

**Table 6:** Model formula and associated AIC values: Part 1

Model	Model formula	AIC
E2ME	Accuracy ~ 1 + (1|Participant) + (1|Stimulus)	1480.1
E2M1	Part 1 Accuracy ~ Condition*Trial Type + (1|Participant) + (1|Stimulus)	1310.8

**Fig. 17:**
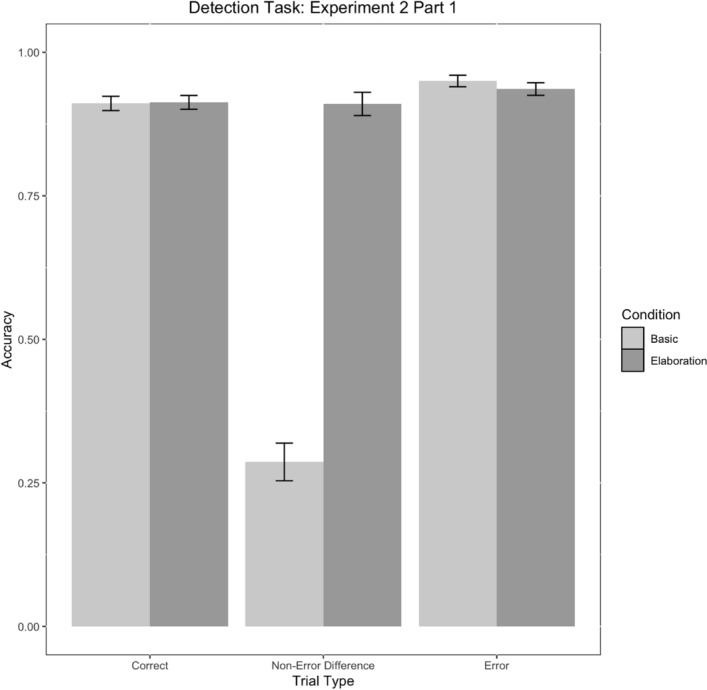
Detection task accuracy by condition and trial type in Experiment 2, Part 1. Error bars represent standard errors

We again observed main effects of condition, trial type, and a condition*trial type interaction (Table 7). Critically, participants in the elaboration condition were again less likely to false alarm to differences as compared to the basic condition (Fig. 17).

**Table 7:** Parameter estimates for model E2M1

Fixed effects	Estimate	*SE*	*No. Obs*	*Z*	*p*	95% CI [LL, UL]
*Parameter*						
Intercept	2.51	0.25	2450	9.91	< .001***	[7.51, 20.29]
Condition	− 0.64	0.14	2450	− 4.67	< .001***	[0.40, 0.69]
Trial Type: Correct versus Other	0.22	0.14	2450	1.59	0.11	[0.95, 1.64]
Trial Type: Difference versus Error	− 1.44	0.30	2450	− 4.83	< .001***	[0.13, 0.43]
Condition*Trial Type: Correct versus Other	0.29	0.05	2450	5.33	< .001***	[1.20, 1.48]
Condition*Trial Type: Difference versus Error	− 1.02	0.11	2450	− 9.06	< .001***	[0.29, 0.45]

Condition Levels: 0 = Elaboration, 1 = Basic. ‘Other’ = Difference and Error trial types combined. No. Obs = Number of observations

***p < .001, **p < .01, *p < .05

Next, we tested whether learners in the elaboration condition also more accurately identified differences when tested on a second bag for which they did not receive elaboration. The first empty model included a random effect for the intercept for stimulus and revealed that stimulus accounted for 27% of the variation in detection accuracy. The second empty model included a random effect for the intercept for participant, revealing that 10% of the variation in accuracy was accounted for by this random effect. Random intercepts for stimulus and participant were therefore retained in the model predicting detection accuracy.

We added a fixed effect of condition and its interaction with trial type to test whether learners who received elaboration in Part 1 of the experiment exhibited greater detection accuracy in Part 2 and whether this pattern varied across trial type. This model (E2M2, Table 8) was a better fit for the data than the null model based on AIC and log likelihood value comparisons ((*χ*^2^ (5) = 66.5, *p* < .001).

**Table 8:** Model formulas and associated AIC values: Part 2.1

Model	Model formula	AIC
E2ME2	Accuracy ~ 1 + (1|Participant) + (1|Stimulus)	2010.4
E2M2	Part 2 Accuracy ~ Condition*Trial Type + (1|Participant) + (1|Stimulus)	1954.0
E2ME3	Accuracy ~ 1 + (1|Participant)	4201.5
E2M3	Part 2 Accuracy ~ Condition*Experiment Part + (1|Participant)	4112.7

**Fig. 18:**
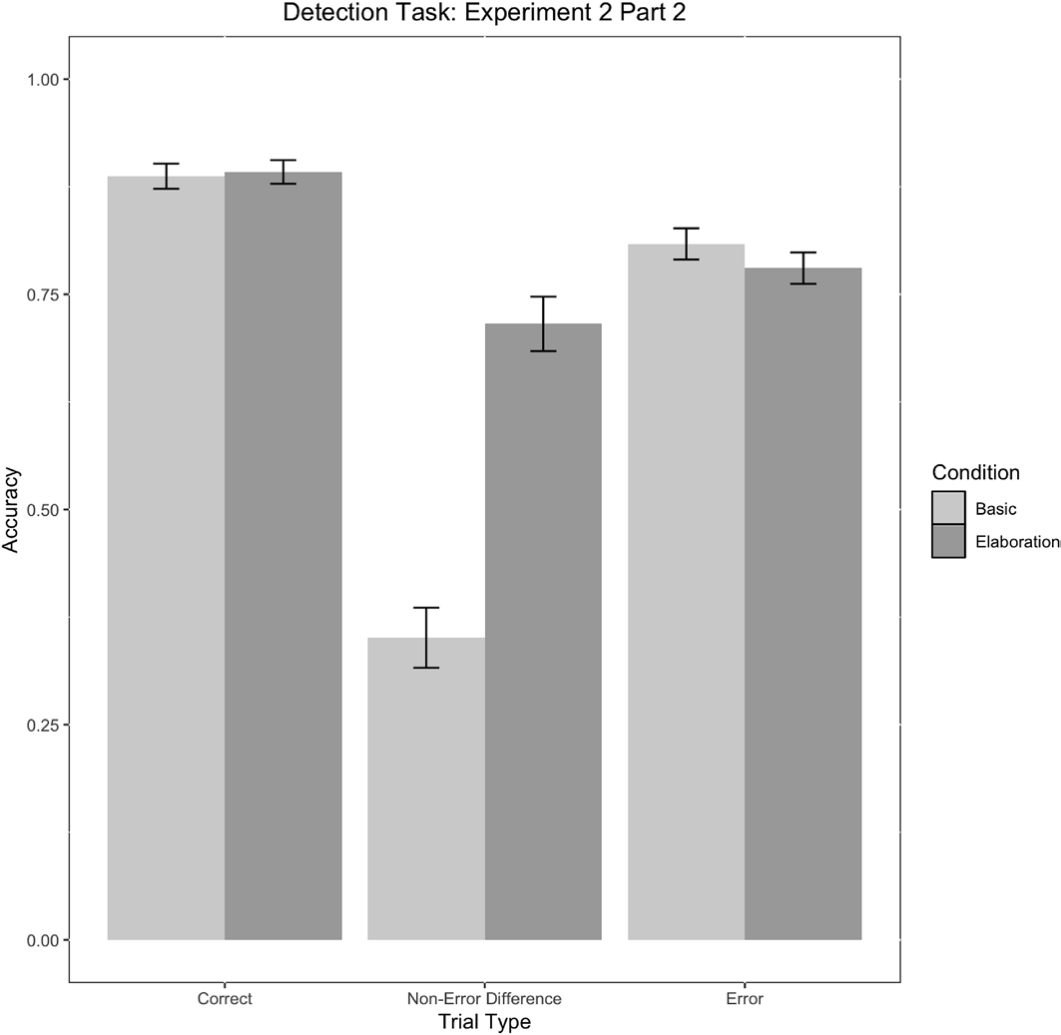
Detection task accuracy by condition and trial type in Experiment 2, Part 2. Error bars represent standard errors

Again, we found that those who had received elaboration in Part 1 were less likely to false alarm to differences compared to the basic condition (Table 9, Fig. 18).

**Table 9:** Parameter estimates for model E2M2

Fixed effects	Estimate	*SE*	*No. Obs*	*Z*	*p*	95% CI [LL, UL]
Parameter						
Intercept	1.57	0.23	2342	6.91	< .001***	[3.08, 7.51]
Condition	− 0.27	0.11	2342	− 2.57	< .05*	[0.62, 0.94]
Trial Type: Correct versus Other	0.49	0.14	2342	3.57	< .001***	[1.25, 2.13]
Trial Type: Difference versus Error	− 0.77	0.27	2342	− 2.88	< .01**	[0.28, 0.78]
Condition*Trial Type: Correct versus Other	0.12	0.04	2342	2.73	< .01**	[1.03, 1.23]
Condition*Trial Type: Difference versus Error	− 0.50	0.08	2342	− 6.68	< .001***	[0.52, 0.70]

Condition Levels: 0 = Elaboration, 1 = Basic. ‘Other’ = Difference and Error trial types combined. No. Obs = Number of observations

***p < .001, **p < .01, *p < .05

Lastly, we were interested in whether learners in the elaboration condition also performed better in Part 2 of the experiment. We estimated multilevel models to compare whether Part (1 or 2) predicted accuracy and whether this varied between the basic condition, who did not receive elaboration in either part, versus the elaboration condition, who received elaboration in part 1 only. We estimated an empty model that only included a random intercept for participant, given that unique stimuli were used across the two parts of the experiment. We found that participant accounted for 12% of the variation in detection accuracy and was therefore retained in the model predicting detection accuracy.

We added a fixed effect of condition and its interaction with experiment part (i.e., phase). This model (E2M3, Table 8) was a better fit for the data than the null model based on AIC and log likelihood value comparisons (*χ*^2^ (3) = 94.8, *p* < .001).

**Fig. 19:**
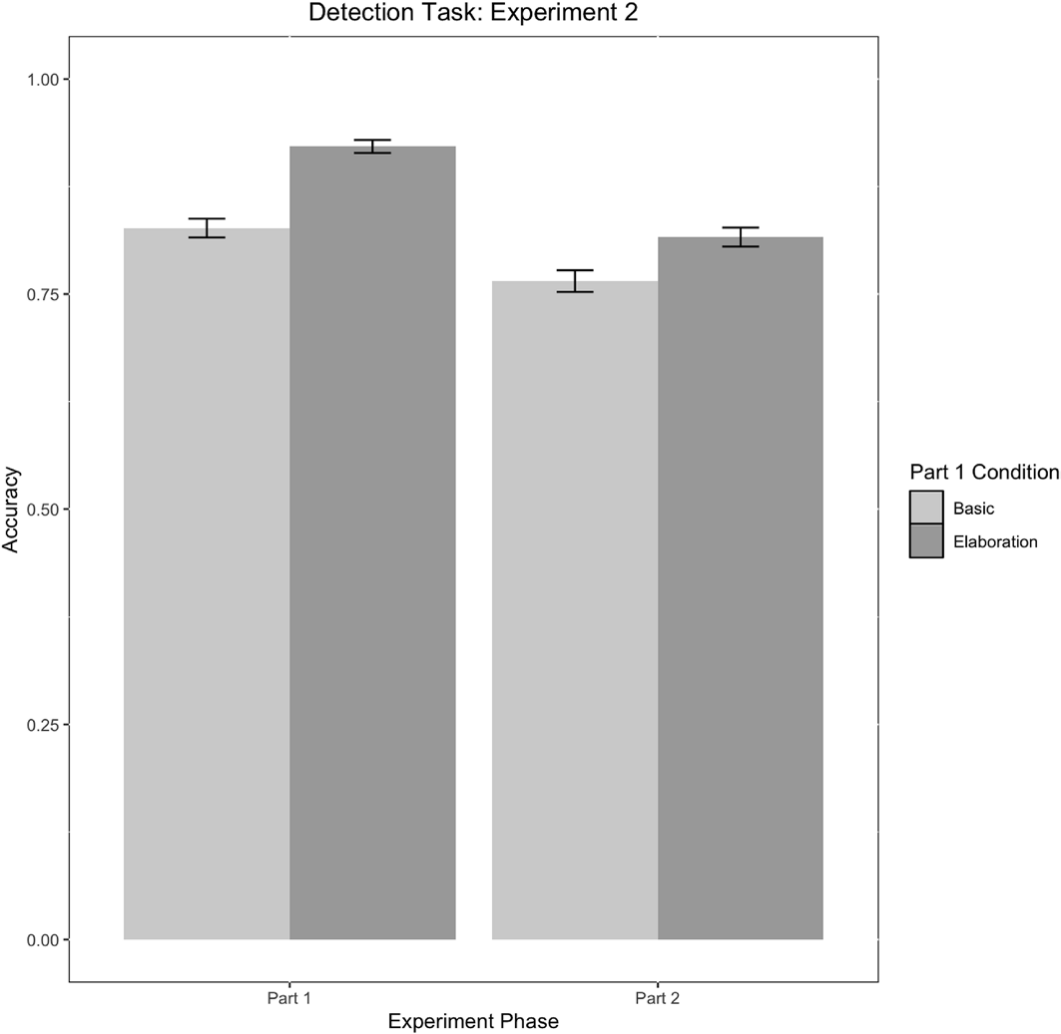
Detection task accuracy by condition and experiment phase in Experiment 2. Error bars represent standard errors

We observed main effects of condition, part, and a condition*part interaction (Table 11). Participants in the elaboration condition were more accurate than the basic condition across both parts of the experiment, and they were most accurate in Part 1 (Fig. 19). Although learners in the Elaboration condition still perform better in Part 2, there is less of a difference in accuracy magnitude between the basic and elaboration conditions.

**Table 11:** Parameter estimates for model E2M3

Fixed effects	Estimate	*SE*	*No. Obs*	*Z*	*p*	95% CI [LL, UL]
*Parameter*						
Intercept	1.63	0.12	4802	13.91	< .001***	[4.04, 6.39]
Condition	1.03	0.18	4802	5.65	< .001***	[1.96, 4.02]
Part	− 0.37	0.11	4802	− 3.58	< .001***	[0.56, 0.85]
Condition*Part	− 0.66	0.17	4802	− 3.85	< .001***	[0.37, 0.72]

Condition Levels: 0 = Elaboration, 1 = Basic. No. Obs = Number of observations

***p < .001, **p < .01, *p < .05

## Discussion

In Experiment 2, we replicated findings from Experiment 1, suggesting that receiving elaboration resulted in more accurate rejection rates of non-error differences at test. This finding is particularly valuable given that we again found that learners in the basic condition performed below chance at correctly rejecting non-error differences. As in Experiment 1, elaboration did not aid learners’ ability to identify correct or error models, though accuracy approached ceiling across both conditions. Importantly, we found that receiving elaboration about the first model transferred to aid performance on a second model: Learners who received elaboration during Part 1 of the experiment also performed better during Part 2, even when they did not receive direct elaboration about errors and differences. That said, there was less of a difference in accuracy magnitude between the two conditions in Part 2. We offer a couple possibilities as to why this result may have occurred. First, it is possible that learners in the elaboration condition independently produced their own elaborations to aid task performance, but the effect was not as strong as the direct elaboration provided in Part 1. Further, it is possible that learners in the basic condition spontaneously produced elaborations to aid learning, though it is unknown. Taken together, our results support the notion that providing learners with additional explanation about what constitutes differences and why they are unimportant to error outcomes can subsequently help to mitigate false alarming.

## General discussion

In two experiments, we assessed whether receiving elaboration about errors and differences aided learners’ ability to accurately detect errors at test. Though elaboration did not aid hit rates on correct and error models, we note that accuracy across both experiments was high overall. In this study, we were particularly interested in whether elaboration mitigated false alarming, or falsely detecting an error when there was not one present. This question has applied implications, where minor variations across payloads may be misinterpreted as meaningful and wrongfully flagged as an issue in military contexts. To experimentally investigate this scenario, we strategically designed models that contained both errors and differences, or visually dissimilar properties within the models that did not pose problems but could provide visually salient and capture attention (Rensink, 2002).

Findings from both experiments suggested that participants were able to successfully learn the components that comprised a correctly-rigged bag model, and elaboration about why a difference in a model was not an error did in fact result in learners more accurately rejecting non-error differences at test. This finding is important, given that across both experiments, learners in the basic condition, who did not receive additional elaboration, performed below chance at correctly rejecting non-error differences at test. In Experiment 2, we expanded on these findings by determining that elaboration on one model could transfer to support error detection performance on a second model. Collectively, these findings provide insight on how training can be optimized to alleviate false alarm tendencies among operators. Namely, drawing attention to salient visual features or “differences” and explaining why they are not consequential may prompt learners to effectively disqualify them in the future.

## Data Availability

Not applicable.
